# Immunophenotyping and transcriptional profiling of *in vitro* cultured human adipose tissue derived stem cells

**DOI:** 10.1038/s41598-018-29477-5

**Published:** 2018-07-27

**Authors:** Alina Mieczkowska, Adriana Schumacher, Natalia Filipowicz, Anna Wardowska, Maciej Zieliński, Piotr Madanecki, Ewa Nowicka, Paulina Langa, Milena Deptuła, Jacek Zieliński, Karolina Kondej, Alicja Renkielska, Patrick G. Buckley, David K. Crossman, Michael R. Crowley, Artur Czupryn, Piotr Mucha, Paweł Sachadyn, Łukasz Janus, Piotr Skowron, Sylwia Rodziewicz-Motowidło, Mirosława Cichorek, Michał Pikuła, Arkadiusz Piotrowski

**Affiliations:** 1 Faculty of Pharmacy, Medical University of Gdansk, Gdansk, Poland; 2 Department of Embryology, Faculty of Medicine, Medical University of Gdansk, Gdansk, Poland; 3 Department of Clinical Immunology and Transplantology, Medical University of Gdansk, Gdansk, Poland; 4 Department of Clinical Anatomy, Medical University of Gdansk, Gdansk, Poland; 5 Department of Surgical Oncology, Medical University of Gdansk, Gdansk, Poland; 6 Department of Plastic Surgery, Medical University of Gdansk, Gdansk, Poland; 7 GMI Genomics Centre, Genomics Medicine Ireland, Dublin, Ireland; 8 Heflin Center for Genomic Sciences, University of Alabama at Birmingham, Birmingham, Alabama USA; 9 Laboratory of Neurobiology, Department of Molecular and Cellular Neurobiology, Nencki Institute of Experimental Biology PAS, Warsaw, Poland; 10 Department of Biochemistry, Faculty of Chemistry, University of Gdansk, Gdansk, Poland; 11 Laboratory for Regenerative Biotechnology, Gdansk University of Technology, Gdansk, Poland; 12 MedVentures company sp. z o.o., Poznań, Poland; 13 Department of Molecular Biotechnology, Faculty of Chemistry, University of Gdansk, Gdansk, Poland; 14 Department of Biomedicinal Chemistry, Faculty of Chemistry, University of Gdansk, Gdansk, Poland; 15 Present Address: Laboratory of Tissue Engineering and Regenerative Medicine, Department of Embryology, Faculty of Medicine, Medical University of Gdansk, Gdansk, Poland

## Abstract

Adipose-derived stem cells (ASCs) have become an important research model in regenerative medicine. However, there are controversies regarding the impact of prolonged cell culture on the ASCs phenotype and their differentiation potential. Hence, we studied 10 clinical ASCs replicates from plastic and oncological surgery patients, in six-passage FBS supplemented cultures. We quantified basic mesenchymal cell surface marker transcripts and the encoded proteins after each passage. In parallel, we investigated the differentiation potential of ASCs into chondrocytes, osteocytes and adipocytes. We further determined the effects of FBS supplementation and subsequent deprivation on the whole transcriptome by comprehensive mRNA and miRNA sequencing. Our results show that ASCs maintain differentiation potential and consistent profile of key mesenchymal markers, with apparent expression of distinct isoforms, in long-term cultures. No significant differences were observed between plastic and oncological surgery cohorts. ASCs in FBS supplemented primary cultures are almost committed to mesenchymal lineages as they express key epithelial-mesenchymal transition genes including early mesenchymal markers. Furthermore, combined mRNA/miRNA expression profiling strongly supports a modulatory role for the miR-30 family in the commitment process to mesenchymal lineages. Finally, we propose improvements to existing qPCR based assays that address alternative isoform expression of mesenchymal markers.

## Introduction

Adipose-derived stem cells have recently become a widely and intensively studied research tool, mainly due to their multidirectional biological activity and broad scope of potential therapeutic application. Currently, many clinical trials utilizing ASCs in the treatment of chronic wounds, inflammatory and cardio-vascular diseases as well as bone and cartilage reconstruction, are taking place worldwide^1,2^. These cells have also become an important research model for the investigation of biologically active compounds and potential therapeutics. The practical application of ASCs requires not only the cells isolation from the tissue, but also continuous cell culture lasting usually to the 2^nd^ passage (with the highest proliferative index in the P2)^3^. However, when a higher number of cells is needed, the cell culture has to be prolonged until the 5^th^ or even 6^th^ passage. Therefore, there is a critical need to assess the long-term culture influence on the phenotype and differentiation potential of ASCs. Recent literature suggest that long-term cell culture does not cause any chromosome aberrations^4^. Nevertheless, the debate regarding the impact of cell culture on the characteristic ASCs markers and their differentiation potential tend to constantly emerge^2^.

Even though, fetal bovine serum (FBS) is a frequently used supplement in human cell cultures, a current trend in basic, pre-clinical and therapeutic applications is to avoid the addition of this component^5,6^. It is in part due to the fact that FBS creates a transmission risk of potentially infectious agents to the cell preparation^7,8^. Furthermore, FBS contains a natural mix of growth factors, hormones, nutrients and many uncharacterized components, causing unspecific cell activation (e.g. proliferation, differentiation)^9^. Therefore, FBS is often removed for a short period of time during cell culture (e.g., testing drug candidates) or shortly before the clinical application of cellular product^9,10^. Hence, it is critical to evaluate the impact of short-term serum deprivation of FBS on the adipose-derived stem cells activity^2,11^.

**Figure 1:**
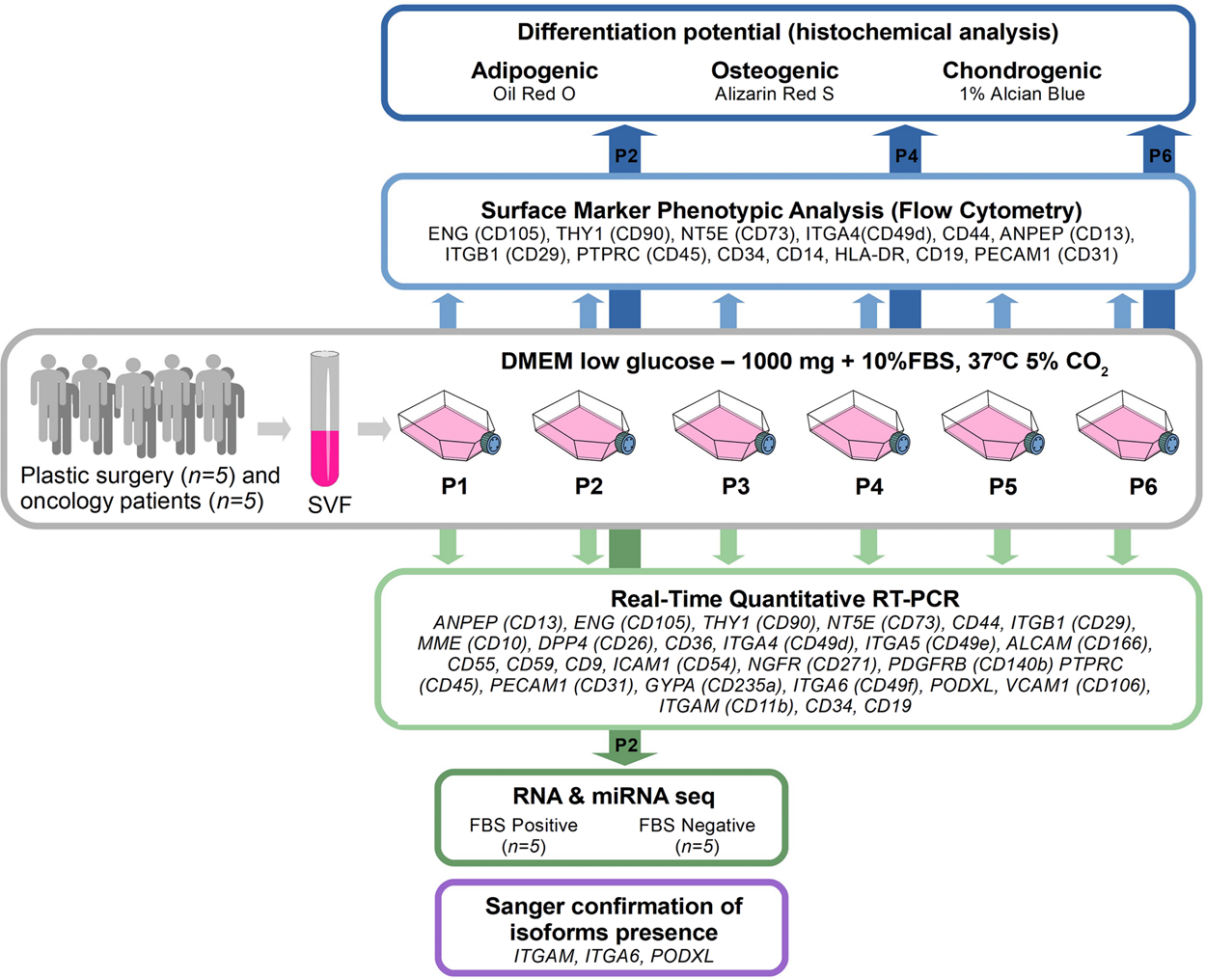
Schematic outline of the experimental workflow. ASCs were isolated from human subcutaneous adipose tissue collected from plastic surgery (*n* = 5) and oncological patients (*n* = 5). Stem cells obtained from SVF (stromal vascular fraction) were cultured up to the 6th passage. The study consisted of five main phases: (*i*) ASCs immunophenotype determination using Flow Cytometry Analysis for 13 surface (P1 - P6), (*ii*) examination of ASCs potential to differentiate to adipocytes, osteocytes and chondrocytes after P2, P4 and P6, (*iii*) Real-Time Quantitative RT-PCR analysis for 27 ASCs positive and negative “stemness” markers made after each passage (P1–P6), (*iv*) RNA-seq and miRNA-seq analysis made for 5 replicates after P2 in two experimental conditions: standard (FBS Positive) and serum-deprived (FBS Negative), (*v*) Sanger sequencing verification of RNA-seq analysis for three transcripts (*ITGAM*, *ITGA6*, *PODXL*) for which significant isoform switching was identified. Further details of the experimental procedure are described in the materials and method section. This figure utilizes modified clipart elements from https://openclipart.org that are covered by Creative Commons Zero 1.0 Public Domain License (http://creativecommons.org/publicdomain/zero/1.0/ and https://openclipart.org/share).

Here, we studied ASCs that were derived from clinical samples of adipose tissue, in long-term, six-passage, FBS supplemented cultures (Fig. 1). We evaluated mesenchymal cell surface markers after each passage with two independent approaches: protein and mRNA quantitation. In parallel, we investigated differentiation potential of ASCs into chondrocytes, osteocytes and adipocytes, after every second passage (P2, P4, P6). In the course of this evaluation, we also compared the characteristics of ASCs from plastic surgery and oncological surgery patients. We further determined the effects of FBS supplementation and subsequent deprivation (P2) on the whole transcriptome, including comprehensive mRNA and miRNA profiling. Finally, we developed a qPCR based assay that addresses alternative isoform expression of mesenchymal cell surface markers, as demonstrated in this study.

## Results

### ASCs exhibit stable expression of key mesenchymal surface markers on protein and transcript level during long-term cell culture

**Figure 2:**
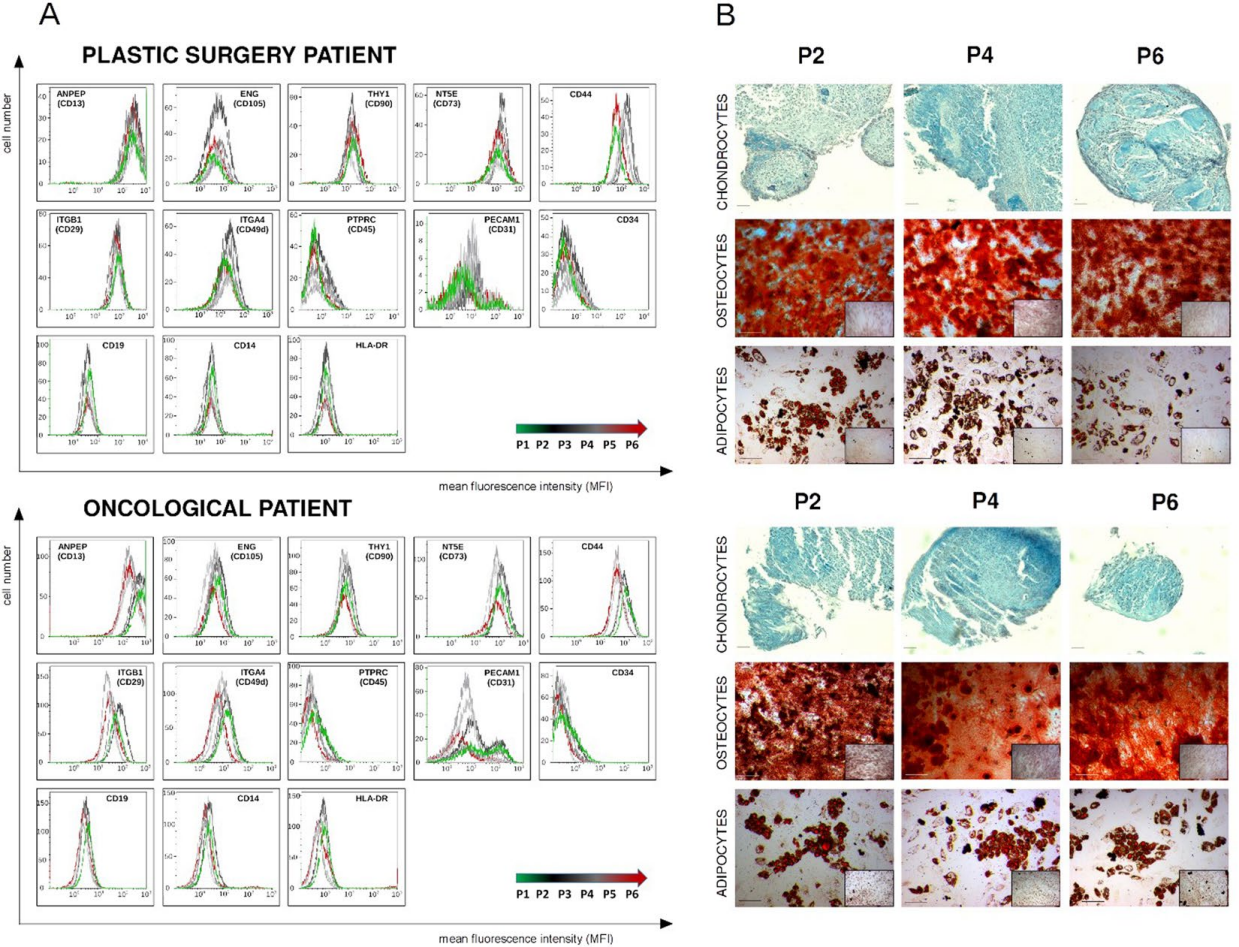
Phenotype identification (**A**) and assessment of differentiation potential (**B**) of ASCs originating from plastic and oncological surgery patients. (**A**) Phenotyping of ASCs standard and supplementary markers using flow cytometry (FCM). FCM plots are shown for two representative patients: plastic and oncological surgery. The X axis represents mean fluorescence intensity (MFI), Y axis - cell number. (**B**) Representative example of histochemical analysis of ASCs differentiation into chondrocytes, osteocytes and adipocytes from plastic surgery and oncological patients. Differentiation assays were performed after 2^nd^ (P2), 4^th^ (P4), and 6^th^ (P6) passage, oil red O, alizarin red S and 1% alcian blue staining were used to confirm adipocytes, osteocytes and chondrocytes differentiation respectively.

**Figure 3:**
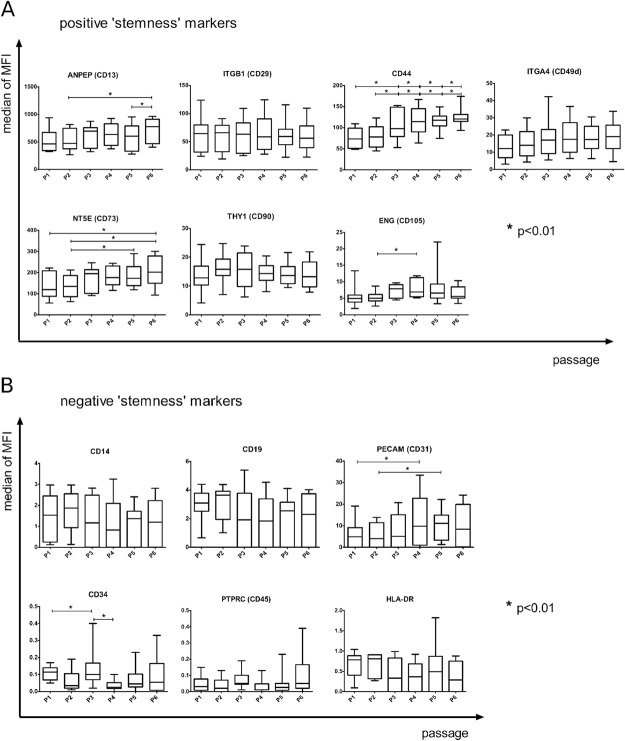
The phenotypic analysis of cultured ASCs. The results are based on 10 biological replicates, including 5 plastic surgery and 5 oncological surgery patients. Flow cytometry assessment of both positive (**A**) and negative (**B**) ASCs markers showed statistically significant changes in the expression of the following positive markers: ANPEP (CD13), CD44, NT5E (CD73) and ENG (CD105); and two negative surface markers of ASC – PECAM1 (CD31) and CD34 (all marked with*). The X axis represents the passage number (from P1 to P6), whereas the Y axis displays the median fluorescence intensity (MFI) of cells expressing particular surface marker. The Wilcoxon signed-rank test was used to compare the dynamics of changes between passages. Statistical significance was accepted when P was ≤ 0.01. Center lines denote the median, box limits indicate the 25th and 75th percentiles; whiskers represent the maximum and minimum of the acquired values.

As shown by flow cytometry, the majority of the analyzed surface markers displayed no significant changes in expression throughout the 6-week/6-passage ASCs culture, confirming stable phenotype of ASCs (Figs 2A and 3A,B). Furthermore, three positive surface markers showed a favorable increase of expression towards the final passages (Fig. 3A,B). Specifically, CD44 expression visibly increased throughout the duration of the cell culture. Another two positive markers, ANPEP (CD13) and NT5E (CD73) displayed a statistically significant increase from P2/P3 to P6. At the same time, only two negative “stemness” markers showed unfavorable fluctuations of expression between passages. PECAM1 (CD31) slightly increased during the cell culture, reaching the maximum expression at P5. CD34, a recognized negative marker of ASCs, showed significant, albeit isolated, increase at P3. Nonetheless, its expression remained constantly low throughout the passages.

**Figure 4:**
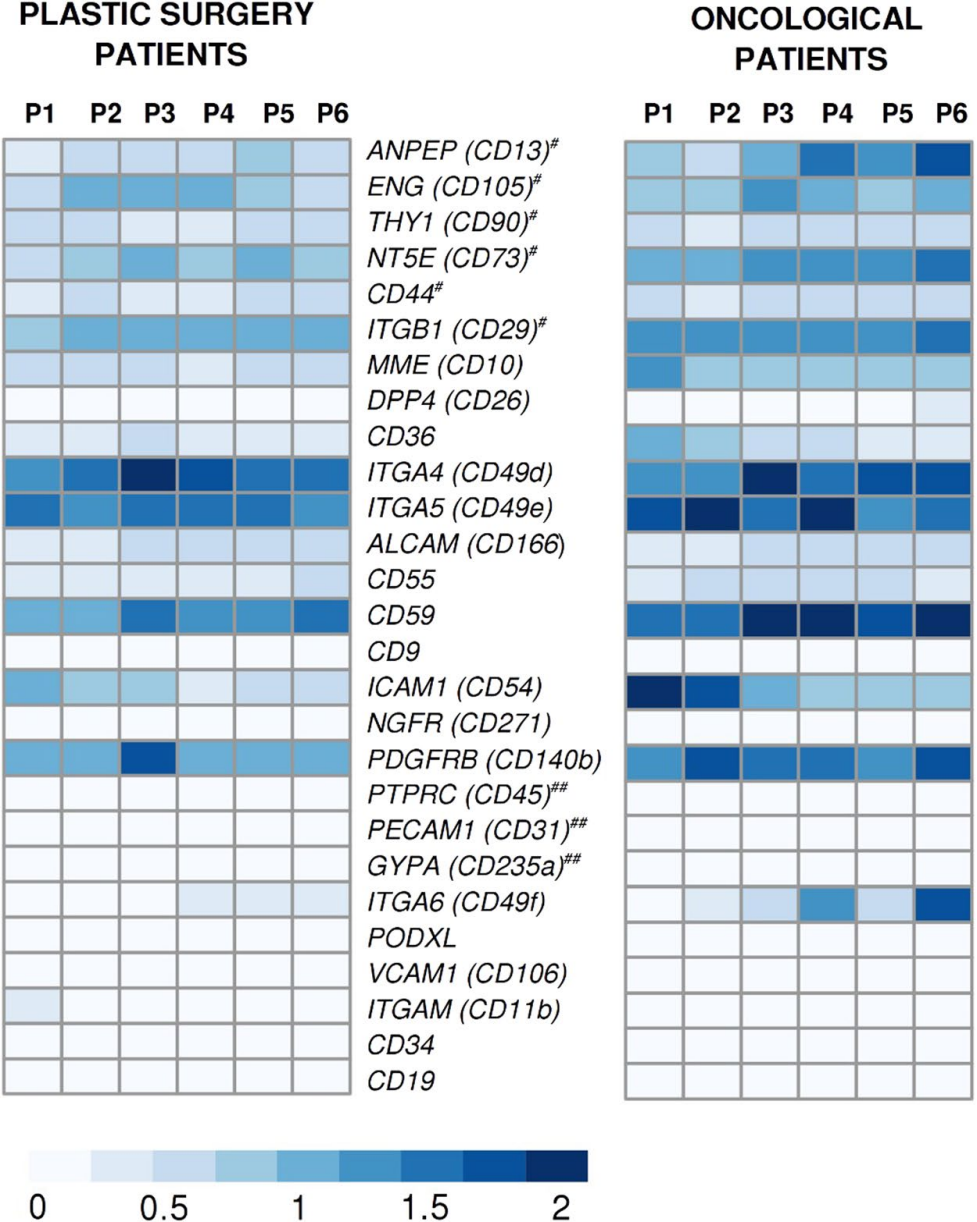
Quantitative PCR gene expression heat maps of primary positive (#) and primary negative (##), secondary positive and negative, non-classical positive and negative ASCs markers. Customary CD nomenclature is supplemented with the official gene names in parentheses. Color intensity depicts transcript quantity, i.e. median expression of target to geometric mean of *HPRT1*, *RPLP0* and *RPL13A* reference genes expression(T/R values) from white (“0”) to dark blue (“2”). Median value from 5 biological replicates in each group is shown for every passage (P1-P6) for two examined groups of patients from plastic (5 individuals) and oncological surgery (5 individuals).

In parallel, we developed quantitative PCR assays to measure transcripts of stemness markers (Fig. 4, Supplementary Tables S2 and S3). This extended panel of markers was selected based on consistent reporting from several independent studies^12–17^. The positive and negative ASCs markers were subdivided into main, secondary and non-classical categories according to Bourin *et al*. and Camilleri *et al*.^17,18^. The qPCR evaluation was carried out after each passage throughout the experiment (P1-P6) on ASCs with confirmed identity by cytometry and differentiation tests.

Transcript quantity (median of target to three reference genes-T/R) of the main positive ASCs markers *ANPEP*(*CD13*), *ENG*(*CD105*), *THY1*(*CD90*), *NT5E* (*CD73*), *CD44*, *ITGB1*(*CD29*), as well as secondary positive ASCs markers, *MME* (*CD10*), *DPP4* (*CD26*), *CD36*, *ITGA4* (*CD49d*), *ITGA5* (*CD49e*), *MME* (*CD10*), remained relatively stable during passages (P1-P6). The primary negative ASCs markers, *PECAM1*(*CD31*), *PTPRC* (*CD45*), *GYPA* (*CD235a*), as well as secondary negative markers *ITGA6* (*CD49f*), *PODXL*, *VCAM1* (*CD106*), *ITGAM* (*CD11b*) showed little or no expression of the encoding transcripts in the final passages, albeit noticeable fluctuations were observed in the initial passages. Specifically, among the main negative markers *PECAM1*(*CD31*) was present up to the P2, however at a very low level (median T/R from 0.04 to 0.09). *PTPRC*(*CD45*) and *GYPA*(*CD235a*) were not detected throughout all six passages. Among the secondary negative markers *VCAM1* (*CD106*) showed no expression. *ITGAM* (*CD11b*) was present only up to P3 and at a very low level (median T/R between 0.04 and 0.09), while *PODXL* showed low but consistent expression throughout passages (median T/R from 0.04 to 0.18). At the same time, expression of *ITGA6* (*CD49f*) gradually increased with the passages, although remained relatively low compared to the expression level of the positive markers, with an exception of the final passages in oncological patients (median T/R values from 0.72 to 1.87).

Additionally, we evaluated non-classical, reportedly positive ASCs markers: *ALCAM* (*CD166*), *CD55*, *CD59*, *CD9*, *ICAM1* (*CD54*), *NGFR* (*CD271*), *PDGFRB* (*CD140b*). *ALCAM* (*CD166*) and *CD55* showed relatively stable expression levels throughout the passages. *CD9 and CD59* were also expressed in P1-P6 with marked increase of CD59 towards the final passages (median T/R from 1.15 to 2.23) whereas CD9 remained stable at low levels during subsequent passages (median T/R from 0.1 to 0.33). Conversely, *ICAM1* (*CD54*) showed a steady decrease with subsequent passages (from 2.07 median T/R value in P1 to 0.7 median T/R value in P6) and *NGFR* (*CD271*) displayed low expression that was detectable only in P1 (median T/R value of 0.1). *PDGFRB* (*CD140b*) showed high expression throughout P1–P6. Similarly, we observed inconsistencies in expression levels of one non-classical, reportedly negative ASCs marker *CD34*, that was detectable at low levels up to P3 (median T/R from 0.05 to 0.35). As expected, CD19, a negative marker displayed no expression.

### ASCs from plastic surgery and oncological patients cultured up to 6th passage maintain their differentiation potential

The ASCs differentiating potential determined by histochemical staining followed by semi-quantitative analysis demonstrated that cells could differentiate into adipocytes, osteocytes and chondrocytes up to P6 (Figs 2B and S2). Specifically, in culture with adipogenic medium, ASCs showed adipogenic potential in all passages. The cellular accumulation of lipids estimated by the Oil red staining showed that this potential is maintained up to P6. In culture with osteogenic medium, ASCs maintained the ability to deposit calcium, as detected by Alizarin red staining, up to P6. After culture with chondrogenic medium, histological slides were stained with Alcian blue to visualize the glycosaminoglycans (GAG) secretion by differentiating chondrocytes. In all formed pellets the presence of glycosaminoglycans as blue stained deposits between cells was histochemically confirmed. No significant changes in the potential of ASCs to differentiate into adipocytes, osteocytes, chondrocytes in time of cell culture between two groups of examined patients, was observed (Supplementary Fig. S2). Moreover, evaluation of ASCs population doublings up to P6 showed no statistically significant differences between oncological and plastic surgery patients (Supplementary Fig. S3).

### Comparison of ASCs surface markers from plastic and oncological surgery patients

Biological replicates, i.e. ASCs derived from different individuals, presented a similar pattern of expression of mesenchymal surface markers in qualitative terms (Supplementary Fig. S1A,B). At the same time, notable quantitative inter-replicate differences were observed. These differences were more pronounced in the initial passages of cell cultures and tended to stabilize during the course of subsequent passages. Additionally, possible differences between plastic and oncological surgery cohorts were assessed, however no statistically significant differences were observed between these two groups of patients on both the protein and mRNA levels (Fig. 4, Supplementary Table S3 and Supplementary Fig. S1).

### Whole transcriptome RNA-seq and miRNA-seq analysis of FBS deprivation after P2 indicates lower metabolic and proliferative activity without negative impact on cell viability

**Figure 5:**
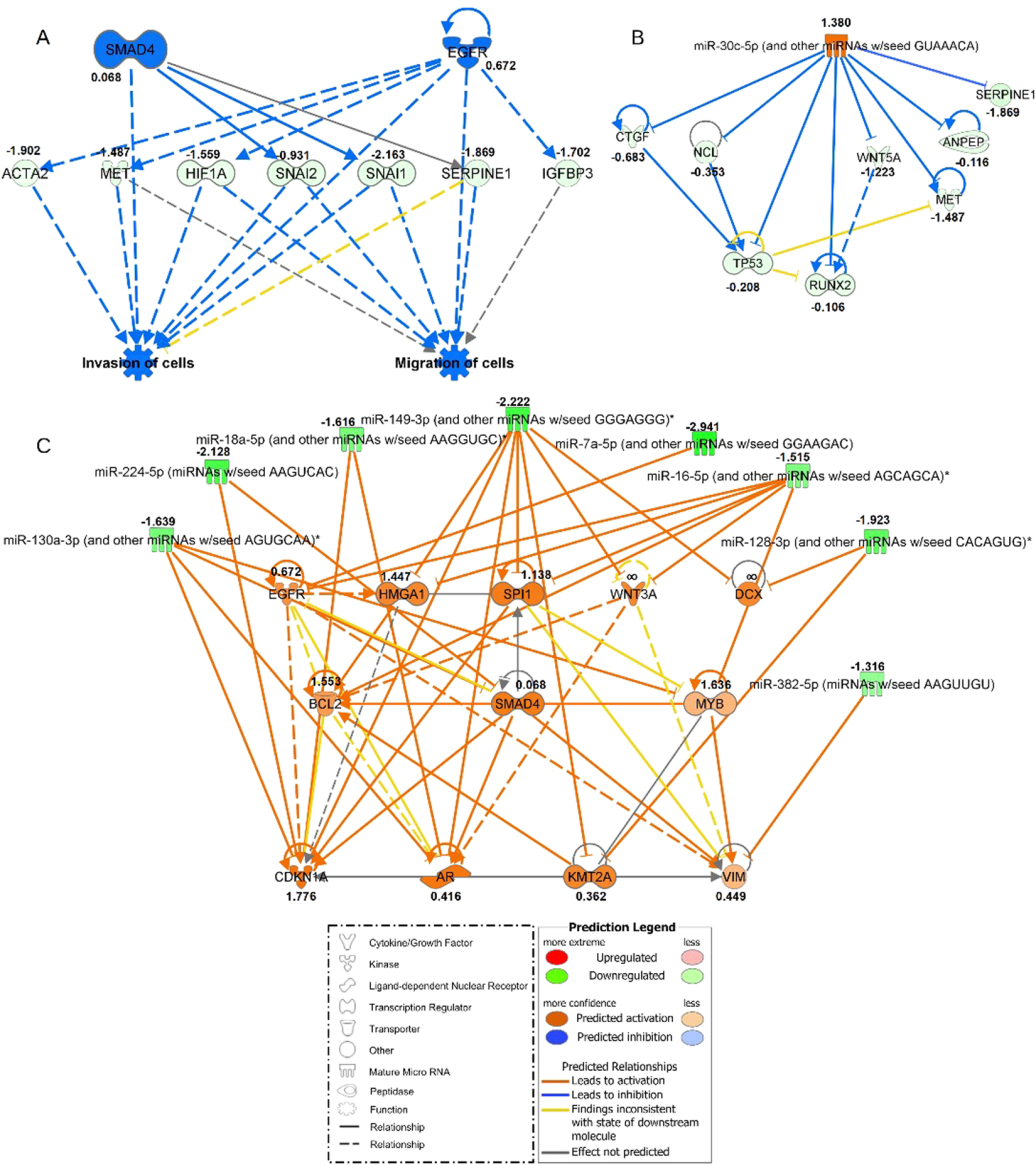
Networks generated in Ingenuity Pathway Analysis (IPA) for ASCs cultured in standard FBS-supplemented and FBS-deprived conditions for five biological replicates (plastic surgery patients, Supplementary Table S1). The values next to the gene names refer to log_2_ fold change of gene expression calculated in the cufflinks package. The shapes and colors of molecules and type of interactions are defined in the graphical legends. (**A**) Top Regulator Effects network based on differentially expressed genes in both experimental conditions with highest consistency score of 3.024. The middle row consists of molecules that are connected to upstream regulators in the top row and downstream functions in the bottom row. (**B**) miRNA-mRNA negative correlation network with upregulated mir-30 family and anti-correlated mRNA targets generated in microRNA Target Filter Tool in IPA. (**C**) miRNA-mRNA negative correlation network with downregulated 8 miRNAs and their upregulated direct targets. miRNAs indicated with a star refer to miRNA families that share common seed sequence with those significantly changed in our dataset.

Differential expression analysis of the transcriptomes was carried out using Cufflinks RNA-seq workflow^19,20^ on independent set of five biological replicates in two experimental conditions: FBS-deprived ASCs culture versus FBS-supplemented ASCs culture, after P2 (Supplementary Table S1). Transcripts that showed significant and consistent differences between conditions in our dataset (FDR corrected p-value, q-value < 0.05) were subjected to further interpretation with Ingenuity Pathway Analysis (IPA). The IPA core expression analysis encompassed known and experimentally confirmed interactions in human tissues and primary cells. These interactions were subsequently evaluated in the context of causal networks related to migration, proliferation, differentiation, developmental processes, expansion, and maturation of adipose-derived stem cells. The IPA core expression analysis indicated 433 analysis ready molecules across observations. Next, the regulator effects algorithm in IPA identified the association between the molecules in our datasets and connect them with upstream regulators and predict downstream effects (Fig. 5A). *SMAD4* and *EGFR* are the likely master regulator for the network with the highest Consistency Score (value 3.024). The identified transcripts which were downregulated included: *ACTA2*, *MET*, *HIF1A*, *SNAI2*, *SNAI1*, *SERPINE1*, *IGFBP3*, which causes the functional effect of inhibition of cells migration and invasion of cells after FBS deprivation (Fig. 5A). Disease and function mode implemented in IPA resulted in the list of several phenomena from which 5 were significantly downregulated (p-score < 0.05 and z-score ≤ −2) (Supplementary Table S4). Deprivation of FBS strongly correlated with the decrease of cellular movement, migration, cytoskeleton and cytoplasm organization, cell-to-cell signaling and interactions, and cell proliferation.

Following RNA-seq, we carried out miRNA-seq analysis for the same set of samples. The microRNA Target Filter tool in IPA allowed us to get an insight into miRNA-mRNA interactions. Filtering settings were focused on experimentally observed relationships in human stem cells and based on opposing expression patterns between miRNA and target mRNA pairs (miRNA-mRNA negative correlation networks); i.e. upregulated miRNA- downregulated mRNA (Fig. 5B and Supplementary Table S5), analogously downregulated miRNA - upregulated mRNA (Fig. 5C and Supplementary Table S5). Only one (miR-30) out of 9 statistically changed miRNAs (Fisher’s exact test p-value < 0.05) showed an increase with parallel decrease of its targets (*CTGF*, *NCL*, *WNT5A*, *ANPEP*, *MET*, *TP53*, *RUNX2*, *SERPINE1*) (Fig. 5B and Supplementary Table S5). Eight miRNAs, namely miR-224-5p, miR-130a-5p, miR-18a-5p, miR-149-3p, miR-7a-5p, miR-16-5p, miR-128-3p, miR-382-5p were downregulated with concurrent upregulation of the target transcripts (namely *EGFR*, *HMGA1*, *SPI1*, *WNT3A*, *DCX*, *BCL2*, *SMAD4*, *MYB*, *CDKN1A*, *AR*, *KMT2A*, *VIM*) (Fig. 5C and Supplementary Table S5).

### Transcriptome sequencing reveals concurrent expression of distinct isoforms of ASCs mesenchymal surface markers

We performed RNA-seq based analysis of isoform expression for mesenchymal surface markers that were originally included in a qPCR panel in the present study. This RNA-seq analysis was carried out with an independent set of five biological replicates (Supplementary Table S1). We evaluated the effect of FBS deprivation on the expression of key mesenchymal surface markers. The majority of markers did not show significant changes in expression upon FBS deprivation. However, statistically significant alterations between the conditions were observed in four positive markers: *ENG* (*CD105*), *THY1* (*CD90*), *DPP4* (*CD26*), *ITGA5* (*CD49e*) and two negative markers (*CD34* and *PODXL*) (Supplementary Fig. S4). Furthermore, we analyzed the proportions of isoforms between different experimental conditions after P2: FBS-supplemented ASCs culture vs FBS-deprived ASCs culture (Supplementary Table S7). Only 5 out of 27 genes expressed a single isoform. The remaining 22 genes showed parallel expression of at least two distinct isoforms. This comparison indicated stable isoform proportions for 11 genes and isoform switching for 11 genes: *ENG* (*CD105*), *ITGAM* (*CD11b*), *MME* (*CD10*), *DPP4* (*CD26*), *CD36*, *ALCAM* (*CD166*), *CD55*, *ITGA6* (*CD49f*), *CD19*, *CD9*, *PODXL*, however the differences between the isoforms usually did not exceed 20%. The most pronounced effect was observed for *ITGAM* (*CD11b*) which exhibited >70% isoform switching between the experimental conditions, while *ITGA6* (*CD49f*) and *PODXL* showed lower fluctuations between isoforms. As *ITGAM* (*CD11b*), *ITGA6 and PODXL* are considered negative ASCs markers, RNA-seq results were alone not conclusive in these cases due to low number of sequencing reads in the pertinent loci. Hence, Sanger sequencing was performed to verify isoform switching in these three genes (Supplementary Fig. S5). In *ITGAM* (*CD11b*), the presence of both reported isoforms NM001145808.1 and NM00632.2 in two experimental condition was confirmed which was consistent with the RNA-seq results (Supplementary Fig. S5 1A–C). Notably, these two isoforms differ only by one codon in exon 14. *ITGA6* showed the presence of two transcript variants in FBS-supplemented and FBS-deprived ASCs culture: NM001079818.2 and NM000210.3 that has an extra exon. Presence of these two isoforms was confirmed by Sanger sequencing (Supplementary Fig. S5 2A–C). In FBS supplemented ASCs culture, *PODXL* was mainly represented by the known NM005397.3 transcript variant (8 exons) with traces of the longer NM001018111.2 (9 exons), while in FBS deprived culture only the first one was detectable (Supplementary Fig. S5 3A–C).

## Discussion

Our study shows that long term primary cultures of ASCs retain their phenotype, based on the expression profile of key mesenchymal surface markers, as measured on the transcript and protein level. It has been implied by several other studies that prolonged culturing of mesenchymal stem cells may adversely affect their phenotype and potential to differentiate^19^. Hence, in guidelines concerning ASCs, there is a noticeable trend towards early termination of ASCs cultures *in vitro*, usually before the second or the third passage^3,15,17,20^. This short term culturing may in turn lead to low, suboptimal cell titer for downstream applications of ASCs. At the same time, comprehensive evaluation of the ASCs phenotype in long term cultures has not been performed so far. Here we demonstrate, with independent methods, that ASCs maintain the correct phenotype at least to the sixth passage in FBS supplemented cultures. Furthermore, this phenotype tends to stabilize in the final passages, as shown by evaluating surface markers after each passage (Figs 3, 4, S1 and Supplementary Table S3).

The observed fluctuations, particularly in early passages, may recapitulate the apparent variability between biological replicates as depicted in Supplementary Fig. S1C,D. Biological differences in ASCs from different individuals have been reported by numerous studies^21,22^. The most pronounced deviations from the normal ASC phenotype have been reported in obese individuals (BMI > 30)^23–25^. As this aberrant phenotype may persist in *in vitro* ASC cultures^21^, we excluded the obese individuals from the present study. Concurrently, we wanted to compare ASCs from healthy, plastic surgery individuals to ASCs from severely ill oncological patients. The main rationale for this comparison was that the latter group may be the main beneficiary of bone or cartilage reconstruction in ASCs based therapy^2,22^. Nonetheless, general health burden as well as aggressive chemo- and radio-therapy might possibly impair ASCs quality from oncological patients^26^. The results of the present study indicate that ASCs from plastic or oncological surgery patients are comparable with regards to the profile of surface markers. These promising results should however be corroborated on a larger cohort of oncological patients and include genotoxicity tests.

The majority of available research data indicate that the differentiation potential of ASCs into chondrocytes, osteocytes and adipocytes does not change during a long-term culture (lasting even up to the 10th-13th passage)^19,27^. But there are also some studies suggesting a decrease in the differentiation abilities of ASCs into osteocytes after the 2nd or 6th passage^3,20^. Our results showed that the examined cells were able to differentiate into the 3 above mentioned cell types, until the 6th passage. Therefore, it is feasible to amplify the ASCs number *in vitro* (till the 6th passage) with subsequent effective production of chondrocytes or osteocytes. Due to the increasing number of patients with osteoarticular disorders, cell therapies based on the above model seem very promising^28^.

In our study we confirmed the presence of both positive: NT5E (CD73), THY1 (CD90), and ENG (CD105) and negative: PTPRC (CD45), CD19, CD14, and HLA-DR markers characteristic for ASCs, according to the available criteria^12^. There are several questions in the literature over the significance of CD34 and PECAM1 (CD31) by ASC^1,29^, therefore we analyzed both of these surface molecules. We observed that ASCs in our study were partially positive for PECAM1 (CD31) and found limited or no expression of the CD34 antigen up to the 6^th^ passage in a cohort of samples that was used for flow cytometry and qPCR analysis (Figs 3 and 4, Supplementary Table S1). However, we observed a spike in expression of CD34 in FBS(+) cultures, in independent set of samples that were used for RNA-seq analysis (Supplementary Fig. S4 and Supplementary Table S1). Hence, the previously reported controversies on CD34 along with our results may indicate high inter individual variability in expression of this marker^30^. At the same time, ANPEP (CD13) and ITGB1 (CD29) revealed stable and high expression, which is in accordance with recent reports reviewed by Mildmay-White (Fig. 3)^31^.

Another aspect of this study that is related to pre-clinical evaluation of ASCs is influence of FBS supplementation and subsequent deprivation. FBS contains a natural mix of growth factors, hormones, nutrients and many uncharacterized components, causing unspecific cell activation (e.g. proliferation, differentiation)^9^. Therefore, FBS is often removed for a short period of time during the cell culture (e.g. testing drug candidates) or shortly before the clinical application of cellular product^9,10^. However, the effect of FBS deprivation on ACSs has not been evaluated in comprehensive way. Thus, we performed sequencing of the entire transcriptome, followed by analysis of gene expression pathways after FBS deprivation in P2. The P2 was selected as it is frequently chosen as an endpoint in studies pertaining to clinical applications of ASCs^3,26^. We found that FBS deprivation makes ASCs enter a state resembling lower metabolic and proliferative activity on the transcriptomic level (Fig. 5A). Several reports have raised concerns that extreme stimulation of ASCs by FBS may cause permanent changes in the response to mitotic checkpoints or may lead to the clonal expansion of cells that have lost the ability to differentiate and do not respond to environmental inhibition^32,33^. Our results suggest that 48-hours FBS deprivation completely abolishes the effect of prior stimulation, as the RNA-seq data shows only remnants of excessive proliferation. On the other hand, analysis of canonical pathways, as implemented in IPA, showed no indications of apoptosis or necrosis. Specifically, FBS deprivation downregulated epithelial-mesenchymal transition protooncogene *MET* and early mesenchymal marker *ACTA2*^34,35^. Further, expression of the paralogous key epithelial-to-mesenchymal transition transcription factors *SNAI1* (Snail) and *SNAI2* (Slug) was present, albeit markedly reduced after FBS deprivation. Both, *SNAI1* and *SNAI2* have evolutionary conserved functions in mesoderm formation, including adipose tissue^36–38^. Similar downregulation was observed for *SERPINE1* and *IGFBP3*. *SERPINE1* encodes plasminogen activator inhibitor-1 (PAI-1) that participates in autocrine regulation of human preadipocyte migration and is also secreted by adult adipocytes^39,40^. *IGFBP3*, insulin-like growth factor binding protein-3, which is the key component in osteogenic differentiation of ASCs was also significantly downregulated after FBS removal^41,42^.

The balanced expression of genes which are characteristic of different mesenchymal lineages reflects the multipotency of ASCs. Numerous studies have demonstrated that ASCs multipotency, proliferation and differentiation are extensively regulated by miRNAs as reviewed by Doo Yeong Kim and Jong-Hyuk Sung^43^. Surprisingly, the coding transcriptome and its regulatory miRNA counterpart have not been evaluated together in ASCs so far. Here, we sequenced and evaluated the entire pool of miRNAs in conjunction with the mRNA expression profile in the same set of biological replicates, i.e. primary ASCs cultures from five donors. Combined mRNA-seq and miRNA-seq analysis was based on the known evidence for interactions of the miRNAs with the target genes as well as an inverse relationship of expression, i.e. miRNA upregulated - mRNA downregulated, miRNA downregulated - mRNA upregulated. This analysis identified 8 downregulated and one upregulated miRNA that are related to adipogenesis (Fig. 5B,C and Supplementary Table S5). Among these miRNAs, the upregulated miR-30 family appeared to regulate several genes including the above described *MET*^44^, *SERPINE1*^45^ as well as *RUNX2*, *WNT5A*^44^ and *CTGF*, which are known modulators of adipogenesis and osteogenesis^42,46–49^. At the same time, increased expression of miR-30 family stimulates chondrogenesis^50^ and adipogenesis with downregulation of osteogenesis^47^. Additionally, decreased expression of miR-224-5p, miR-7-5p, miR-130a-3p and miR-18a-5p states for stimulation of adipogenesis^51^, miR-128-3p inhibits osteogenic differentiation and promotes adipogenesis^52,53^. Overall, the combined expression patterns of mRNA and miRNA indicate that FBS stimulated ASCs are on the brink of commitment to specific mesenchymal lineages (adipogenic, osteogenic or chondrogenic) and these characteristics are partially attenuated following FBS deprivation with a slight shift towards adipogenic lineage.

The whole transcriptome data presented herein also allowed an in-depth analysis of mesenchymal marker transcripts in ASCs. This analysis revealed that concurrent expression of alternative transcript isoforms is common, as 22 out of 27 mesenchymal surface markers were represented by at least two isoforms. The expression of distinct isoforms bears potential consequences for qualitative and quantitative evaluation of mesenchymal surface markers in ASCs. This is due to the monoclonal origin of common antibodies for immunocytochemical techniques which are usually directed against a specific antigenic determinant. At the same time, the exact epitope location is usually not mapped by the manufacturers. This leaves a degree of uncertainty as the capability of the antibodies to capture different protein isoforms is not known. Similarly, available qPCR assays for mesenchymal surface markers in ASCs do not incorporate isoform information. Although, ASCs markers show a generally consistent profile between different studies, apparent heterogeneity does exist as reviewed by Mildmay-White^31^. One of the possible explanations for the observed heterogeneity is expression of different isoforms that are not measured in a consistent way between the studies. Here, we designed and experimentally validated a panel of qPCR assays that is intended to detect the broadest possible spectrum of isoforms (Fig. 4, Supplementary Tables S2 and S3). This panel includes three house-keeping endogenous reference genes that further improve reliability of measurements over assays that utilize only one reference. We propose these solutions as technical improvements overexisting qPCR based assays for the evaluation of mesenchymal surface markers in ASCs.

In conclusion, phenotypic and transcriptional evaluation of key mesenchymal marker genes demonstrate that ASCs maintain their basic properties in long-term, six passage cultures. As part of this evaluation we assessed the influence of FBS supplementation and subsequent deprivation on the entire transcriptome after the second passage, a commonly chosen endpoint in pre-clinical ASCs processing. We combined mRNA and microRNA analysis, for the first time, in a single ASCs study. This pre-clinical assessment showed that ASCs in FBS supplemented primary cultures are almost committed to specific mesenchymal lineages (adipogenic or osteogenic) as they express key epithelial-mesenchymal transition genes including early mesenchymal markers. Furthermore, miRNA expression profiling strongly supports a modulatory role for miR-30 family in the commitment to mesenchymal lineages. These characteristics are partially attenuated following FBS deprivation with a slight shift towards adipogenic lineage and quiescence on transcriptomic level. At the same time, we have found no evidence of apoptosis or necrosis and the ASCs retained expression of their basic surface markers. Our results also indicate that ASCs from plastic or oncological surgery patients are comparable with regards to the profile of surface markers, although inter-individual differences do exist, highlighting the need to include biological replicates in similar studies involving primary ASCs cultures. In summary, the ASCs subjected to FBS supplementation-deprivation cycle do not display features that would preclude their clinical applications. Finally, we propose technical improvements to existing qPCR based assays for the evaluation of mesenchymal surface markers in ASCs. These solutions include a panel of qPCR assays that are intended to reliably quantify the broadest possible spectrum of mesenchymal surface marker isoforms as well as additional reference genes.

## Materials and Methods

### Isolation and Culture of ASCs

Human subcutaneous adipose tissue was sampled from plastic (n = 5) and oncological (n = 5) surgery patients (Supplementary Table S1). The procedure was approved by the Independent Bioethics Commission for Research of the Medical University of Gdansk (NKBBN/387/2014) and written informed consent was obtained from the patients prior to surgery. All experiments were performed in accordance with relevant guidelines and regulations. The isolation of adipose-derived stem cells (ASCs) was based on a standard protocol^54^. Briefly, following enzymatic digestion and erythrocyte lysis, the cells were suspended in culture medium Dulbecco’s modified Eagle medium (DMEM low glucose-1000 mg) supplemented with 10% fetal bovine serum FBS (Sigma-Aldrich, Saint Louis, Missouri, USA). After 24 hr in culture condition (37 °C, 5% CO_2_) non-adherent and non ASCs cells were removed^1^. ASCs were seeded on Primaria flasks (Corning, USA) at a density of 375 000 cells/75 cm^2^ flask. The primary cell culture was named culture P0 (cells before passage). After the first cleaning passage and the six following passages, cultured cells were named P1-P6, respectively. Medium was replenished 24 h after each passage, in order to remove unattached cells, and then every 2 days. Subsequent passages were performed at 70–80% cell confluence and cells were trypsinisedwith 0.25% trypsine/EDTA solution (Sigma-Aldrich, Saint Louis, Missouri, USA), with 7 days interval between passages.

In order to assess the influence of serum-deprivation on the transcriptomic profile of ASCs, including stemness markers, cells after the 2nd passage (P2) were seeded and cultured for 24 h in media supplemented with 10% FBS (adherence). After 24 h the media were changed, and cells were cultured in two different conditions: (1) “serum-deprived condition” −24 h incubation with 5% of FBS (cell adaptation) followed by FBS deprivation (0% FBS) for next 48 h; (2) “standard condition” −72 h incubation with 10% FBS. After this time cells were trypsynised, collected, centrifuged and the pellet was snap frozen for transcriptomic analysis.

### Surface Marker Phenotypic Analysis (Flow Cytometry)

After each passage (P1–P6), the analysis of core surface markers was performed according to the international criteria for ASCs identification using flow cytometry: positive “stemness” markers – ENG (CD105), NT5E (CD73), THY1 (CD90), negative “stemness” markers – PTPRC (CD45), CD34, CD14, CD19 and HLA-DR^12^. For phenotypic analysis, we expanded the minimal “stemness” criteria by introducing additional positive: ANPEP (CD13), ITGB1 (CD29), CD44, CD49d and negative PECAM1 (CD31) surface markers as reviewed by Mildmay-White^31^. Cells from subsequent passages (P1-P6) were trypsinisedwashed and stained with monoclonal antibodies PTPRC (CD45) (HI30 clone/Pacific Orange; LifeTechnologies, Carlsbad, USA), CD34 (4H1 clone/eFluor® 450; eBioscience, Vienna, Austria), ENG (CD105) (SN6 clone/eFluor 450; eBioscience, Vienna, Austria), THY1 (CD90) (5E10 clone/FITC; eBioscience, Vienna, Austria), NT5E (CD73) (AD2 clone/PE; eBioscience Vienna, Austria), ANPEP (CD13) (WM-15 clone/APC; eBioscience Vienna, Austria), ITGA4 (CD49d) (9F10 clone/PE; Becton Dickinson, Franklin Lakes, New Jersey, USA), CD44 (G44–26 (C26) clone/PE eFluor 610; Becton Dickinson, Franklin Lakes, New Jersey, USA), ITGB1 (CD29) (MAR4 clone/APC; BD Bioscience Vienna, Austria), PECAM1 (CD31) (WM-59 clone/APC-eFluor 780; eBioscience, Vienna, Austria), CD19 (HIB19 clone/V500; Becton Dickinson, Franklin Lakes, New Jersey, USA), CD44 (G44–26 (C26) clone/PE eFluor 610; Becton Dickinson, Franklin Lakes, New Jersey, USA), CD14 (MφP9 clone/V500; Becton Dickinson, Franklin Lakes, New Jersey, USA), HLA-DR (G46–6 clone/V500; Becton Dickinson, Franklin Lakes, New Jersey, USA). Cells were incubated with antibodies for 30 min at room temperature (RT). After incubation, step cells were washed with PBS one more time and processed with the BD LSRFortessa flow cytometer and analyzed with Diva Software (BD Bioscience, USA). All antibodies used in the study were certified for flow cytometry analyses.

### Adipogenic and Osteogenic Differentiation

For adipogenic and osteogenic differentiation, cells from passages: 2 (P2), 4 (P4), 6 (P6) were cultured in 96-well plates containing 5 × 10^3^ cells per well in 200 µl differentiating media (StemProAdipogenesis Differentiation kit A100070-01, StemPro Osteogenesis Differentiation kit A100072-01, Gibco by Life Technology). The differentiation tests were performed for 8 biological replicates, as indicated in Supplementary Table S1. As a control cells cultured in MesenPRO RS medium (cat. 12746012, Gibco by Life Technology) were used. The medium was changed twice per week and the cells were induced for two weeks. After two weeks, the medium was removed and the cells were washed in PBS and fixed with 4% formaldehyde for 30 min. Oil Red O (Sigma, cat. O0625) and Alizarin Red S (Sigma, cat. A5533) staining was used to confirm adipocytes and osteocytes differentiation, respectively, following manufacturer’s instruction. The cells were observed using Phase-contrast fluorescent microscope (Zeiss, Oberkochen, Germany) and photographed (Zeiss, AxioVision Software, Oberkochen, Germany). Adipocytes were identified as cells with red–stained lipid vesicles. Osteocyte activity was identified as red-staining calcium deposits in the medium^55,56^.

### Chondrogenic Differentiation

Chondrogenesis differentiation was assessed by micropellet formation. The differentiation tests were performed for 8 biological replicates, as indicated in Supplementary Table S1. In total, 5 × 10^5^ cells from passages were placed in a 15 ml conical tube and centrifuge at 1500 rpm for 5 min. The pellet was cultured in 2 ml of differentiating medium (StemProChondrogenesis Differentiation kit, Gibco by Life Technology) for six weeks, with medium changes twice a week. The pellet was fixed with 4% formaldehyde for 24 hr at RT, dehydrated by applying the ethanol in increasing concentration and embedded in paraffin. Five micron thick tissue histological sections were prepared using a microtome. The paraffin-embedded tissues were deparaffinized, hydrated and stained with 1% Alcian Blue pH 2.5 (Sigma-Aldrich, Saint Louis, Missouri, USA) in 3% acetic acid for 30 min at RT. After staining, the slides were counterstained with Harris’s hematoxylin (Sigma-Aldrich, Saint Louis, Missouri, USA) for 1 min at room temperature. Blue staining indicated synthesis of proteoglycans by chondrocytes^55,56^.

### RNA Isolation and Quality Assessment

ASCs (3–5 × 10^5^ of cells) after each passage (P1-P6) were trypsinised, washed, and snap frozen for transcriptomic analysis. The pellets were stored in −80 °C prior to RNA isolation. RNA was isolated using miRNeasy Mini Kit (Qiagen, Hilden, Germany) according to the original protocol with two modifications: (i) 1-bromo-3-chloropropane was used instead of chloroform to prevent foaming and emulsification and (ii) the elution was carried out with 40 µl of water followed by repeated elution with the entire volume of the original eluate. Quality (RNA Integrity Number - RIN) and concentration of samples was assessed using the Bioanalyzer 2100 Instrument and RNA 6000 Nano Kit (Agilent, Waldbronn, Germany). Samples with RIN values >8.0 were used for further transcriptomic analysis.

### Quantitative PCR analysis of ASCs markers

Total RNA (1 µg) was reverse transcribed into cDNA with Transcriptor First Strand cDNA Synthesis Kit (Roche Diagnostics GmbH, Mannheim, Germany). Evaluation of 27 positive and negative stem cell markers transcripts was conducted with Locked Nucleic Acid probes from the Universal Probe Library – UPL (Roche GmbH Diagnostics, Mannheim, Germany). Real-Time PCR was performed using the LightCycler 480 Probe Master Kit (Roche Diagnostics GmbH, Mannheim, Germany) using Light Cycler 480 v1.5.1 instrument. Reactions were prepared in a total volume of 10 µl. The advanced relative quantification E-method was applied for data analysis, and geometric mean of *HPRT1*, *RPLP0* and *RPL13A* house-keeping genes expression was used as reference (based on stable expression in ASCs from pentaplicate RNA-seq experiments). Gene specific primers and UPLs used in the experiment are listed in Supplementary Table S2.

### RNA- and miRNA Sequencing and Bioinformatic Analysis

RNA-seq/miRNA-seq based transcriptomic comparison between “standard condition” and “serum-deprivation condition” was performed on an independent set of five biological replicates, as indicated in Supplementary Table S1, after passage P2 (see Isolation and Culture of ASC for cell culture details). Total RNA with RIN >8.0 was used for sequencing library preparation. Ribosome reduction kit (Arraystar Inc, Rockville, USA) and SureSelect Strand Specific mRNA library kit (Agilent, Santa Clara, USA) were used according to standard procedures. The cDNA libraries were quantitated using qPCR in a Roche LightCycler 480 with the Kapa Library Quantification Kit for Illumina (Kapa Biosystems, Woburn, USA). Onboard cluster generation and 50 nt paired end RNA sequencing were performed on the Illumina HiSeq. 2500 using the Rapid Run v2 sequencing chemistry and flow cells as recommended by the manufacturer (Illumina Inc., San Diego, USA). Paired end 50 bp sequencing runs were completed and the data was converted to the FASTQ Sanger format using the bcl2fastq converter. Trim Galore was used to remove residual adapter sequences from the reads (https://www.bioinformatics.babraham.ac.uk). Sequencing reads were aligned to the human reference genome assembly (hg19) using TopHat^57,58^. Transcript assembly and estimation of the relative abundances was carried out using Cufflinks^59^ with the following settings: quartile normalization method for the libraries, per-condition dispersion method for replicates and biased correction using canonical hg19 as reference. Cuffmerge and Cuffquant were then used followed by a final comparison analysis in Cuffdiff according to workflow for Cufflinks version 2.2.0 and higher (http://cole-trapnell-lab.github.io/cufflinks/manual/). Transcripts that showed significant and consistent differences between conditions in our dataset (FDR corrected p-value, q-value < 0.05) were subjected to further interpretation with Ingenuity Pathway Analysis. Differentially expressed genes were grouped in functional pathways using Ingenuity Pathway Analysis (IPA Spring Release 2017, Redwood City, USA) on gene level using RefSeq annotation. Additionally, differentially expressed genes (q-value < 0.05) from the above Tuxedo suite pipeline were subjected to side-by-side comparison to the results from the alternative data analysis pipelines, i.e. STAR-Cufflinks or STAR-HTSeq-DESeq. 2 combination (Supplementary Table S8).

miRNA-seq was performed for the same set of five biological replicate experiments using QIAseq miRNA Library Kit (Qiagen, Hilden, Germany) following the manufacturer protocol, starting with 500 ng of input RNA for each sample. Quality control and concentrations of individual libraries were assessed with Bioanalyzer 2100 Instrument and High Sensitivity DNA Kit (Agilent, Waldbronn, Germany). miRNA-sequencing was performed on the Illumina MiniSeq using the MiniSeq High Output Kit (single end, 75 cycles) as recommended by the manufacturer (Illumina Inc., San Diego, USA). The resulting FASTQ files were subjected to analysis using Qiagen online platform (http://ngsdataanalysis.sabiosciences.com/QIAseqmiRNA/). Next, the microRNA Target Filter tool in IPA was used to infer likely miRNA-mRNA interactions. Filtering settings were focused on experimentally observed relationships in human stem cells and based on opposing expression patterns between specific miRNAs (including miRNA families with the same seed sequence) and target mRNA.

### Isoform Analysis

To check the proportions of gene isoforms, RNA-seq data was analyzed as described in the previous section with the modification to the Cufflinks settings, where hg19 reference annotation gtf file and RABT assembly (-g option) were used to identify potentially new genes and isoforms^60,61^. For three negative stemness markers (namely *ITGA6*, *ITGAM* (*CD11b*) and *PODXL*) the presence of various isoforms was confirmed with Sanger sequencing. Primers for selected gene regions allowing to distinguish between particular isoforms were designed (Supplementary Table S6) with universal M13 sequencing adaptors attached to 5′-ends of the forward and reverse primers (M13F: 5′-GTAAAACGACGGCCAGT-3′, M13R: 5′-CAGGAAACAGCTATGAC-3′).

### Statistical analysis

All data was expressed as median ± min-max, unless otherwise indicated.

Nonparametric statistical analysis was used based on the small sample size. Comparison of parameters between all groups was made with the Kruskal-Wallis test, and between two groups (oncological and plastic surgery patients) - with the Mann-Whitney U test. The Wilcoxon signed-rank test was used to compare the dynamics of changes between passages. Statistical significance was accepted when P was ≤ 0.01. All analyzes were performed with Statistica v.12 (Statsoft, Warsaw, Poland) software, graphs were prepared either with Statistica v.12 or GraphPad Prism 5 (GraphPad Software, La Jolla, USA).

### Data availability

Both next generation sequencing (NGS) raw and processed matrices are available in ArrayExpress database (http://www.ebi.ac.uk/arrayexpress) under accession number E-MTAB-6436 (RNA-seq) and E-MTAB-6390 (miRNA-seq).

## Electronic supplementary material

Dataset 1

Dataset 2
