# Supplementary material for: Immunophenotyping and transcriptional profiling of in vitro cultured human adipose tissue derived stem cells
Source: Sci Rep. 2018 Jul 27;8:11339. doi: 10.1038/s41598-018-29477-5 (PMC6063933; doi:10.1038/s41598-018-29477-5)
Supplement: Supplementary file 1 — Dataset 1 [file 41598_2018_29477_MOESM1_ESM.docx]

**Immunophenotyping and transcriptional profiling of *in vitro* cultured human adipose tissue derived stem cells.**

**Alina Mieczkowska^1#^, Adriana Schumacher^2#^, Natalia Filipowicz^1^, Anna Wardowska^3,15^, Maciej Zieliński^3^, Piotr Madanecki^1^, Ewa Nowicka^4^, Paulina Langa^3^, Milena Deptuła^2,15^, Jacek Zieliński^5^, Karolina Kondej^6^, Alicja Renkielska^6^, Patrick G. Buckley^7^, David K. Crossman^8^, Michael R. Crowley^8^, Artur Czupryn^9^, Piotr Mucha^10^, Paweł Sachadyn^11^, Łukasz Janus^12^, Piotr Skowron^13^, Sylwia Rodziewicz-Motowidło^14^, Mirosława Cichorek^2^, Michał Pikuła^3,15*^, Arkadiusz Piotrowski^1*^**

**^#^These authors contributed equally to this work**

**^*^These authors jointly supervised this work, corresponding authors**

**^1^Faculty of Pharmacy, Medical University of Gdansk, Gdansk, Poland**

**^2^Department of Embryology, Faculty of Medicine, Medical University of Gdansk, Gdansk, Poland**

**^3^Department of Clinical Immunology and Transplantology, Medical University of Gdansk, Poland**

**^4^Department of Clinical Anatomy, Medical University of Gdansk, Poland**

**^5^Department of Surgical Oncology, Medical University of Gdansk, Gdansk, Poland**

**^6^Department of Plastic Surgery, Medical University of Gdansk, Poland**

**^7^GMI Genomics Centre, Genomics Medicine Ireland, Ireland**

**^8^Heﬂin Center for Genomic Sciences, University of Alabama at Birmingham, Birmingham, Alabama, USA**

**^9^Laboratory of Neurobiology, Department of Molecular and Cellular Neurobiology, Nencki Institute of Experimental Biology PAS, Warsaw, Poland**

**^10^Department of Biochemistry, Faculty of Chemistry, University of Gdansk, Gdansk, Poland**

**^11^Laboratory for Regenerative Biotechnology, Gdansk University of Technology, Gdansk, Poland**

**^12^MedVentures company sp. z o.o., Poznan, Poland**

**^13^Department of Molecular Biotechnology, Faculty of Chemistry, University of Gdansk, Gdansk, Poland**

**^14^Department of Biomedicinal Chemistry, Faculty of Chemistry, University of Gdansk, Gdansk, Poland**

**^15^Present: Laboratory of Tissue Engineering and Regenerative Medicine, Department of Embryology, Faculty of Medicine, Medical University of Gdansk, Poland**

**Supplementary Tables and Figures – Table of Contents**

**Item no. and title Page**

**Supplementary Table S1:** Patients summary. 3

**Supplementary Table S2:** Summary of quantitative PCR assay design.
(File “Suppl. Info.)

**Supplementary Table S3.** Results of qPCR assessment of ASCs markers for plastic
as well as oncological surgery patient groups. (File “Suppl. Info.)

**Supplementary Table S4:** Top functions and diseases predicted to be altered in ASCs 4
after FBS deprivation compared to ASCs cultured with standard condition with FBS.

**Supplementary Table S5:** Whole transcriptome, mRNA-seq and miRNa-seq analysis
of FBS deprivation after P2 exported from Ingenuity Pathway Analysis (IPA).
(File “Suppl. Info.)

**Supplementary Table S6:** Primers used for *ITGAM, ITGA6* and *PODXL* isoform 5
analysis with amplification products and their length.

**Supplementary Table S7:** ASCs stemness markers isoform analysis results from
RNA-seq experiment based on 5 biological replicates, after 2^nd^ passage (P2), with
and without FBS stimulation. (File “Suppl. Info.)

**Supplementary Table S8:** Differentialy expressed genes between ASCs after FBS deprivation and ASCs cultured with standard condition media with FBS, based on
 biological pentaplicate. (File “Suppl. Info.)

**Supplementary** **Figure S1**: The flow cytometry analysis of the differences between 6
analyzed groups – oncological patients vs. plastic surgery patients.

**Supplementary Figure S2** Quantitative analysis of the multilineage differentiating 8 potential of oncological and plastic surgery patients ASCs.

**Supplementary Figure S3** Cumulative population doublings of ASCs 9

**Supplementary Figure S4**: RNA-seq results comparison of ASCs markers expression 10
with FBS-deprived medium and with FBS supplemented condition.

**Supplementary Figure S5**: Sanger sequencing confirmation of *ITGAM (CD11b)*, 12
*ITGA6* and *PODXL* isoforms for ASCs cultured in absence and presence of FBS

**SUPPLEMENTARY MATERIALS**

**SUPPLEMENTARY TABLES**

**Supplementary Table S1**

| Patient | Category | Age | Sex | BMI^1^ | Diagnosis |
| --- | --- | --- | --- | --- | --- |
| OS1^3,4^ | O | 42 | F | 21 | Liver cancer |
| OS2^3,4^ | O | 68 | F | 29 | Colorectal cancer |
| OS3^4^ | O | 64 | F | 22 | Stomach cancer |
| OS4^3,4^ | O | 65 | M | 24 | Sigmoid colon cancer |
| OS5^3,4^ | O | 71 | F | 29 | Stomach cancer |
| PS1^2,4^ | P | 53 | F | 25 | Excess of thigh skin (bariatric patient) |
| PS2^3,4^ | P | 62 | F | 28 | Breast hypertrophy |
| PS3^3,4^ | P | 44 | F | 29 | Excess of abdomen skin (bariatric patient) |
| PS4^3,4^ | P | 32 | F | 23 | Excess of abdomen skin (bariatric patient) |
| PS5^3,4^ | P | 44 | F | 26 | Excess of abdomen skin (bariatric patient) |
| PS6^2^ | P | 44 | F | 20 | Excess of arm skin (bariatric patient) |
| PS7^2^ | P | 51 | F | 24 | Face malformation |
| PS8^2^ | P | 59 | F | 28 | Breast reconstruction |
| PS9^2^ | P | 47 | F | 26 | Abdomen malformation |

**Supplementary Table S1:** Patients summary. Abbreviations: category O – oncological surgery patient, P – plastic surgery patient, sex F – female, M – male. ^1^ Only patients with BMI<30 were selected for the analysis to avoid possible obesity related bias. ^2^ Material from PS1, PS6, PS7, PS8, PS9 was used for RNA-seq analysis as well as RNA-seq based transcript isoform analysis. ^3^ Material from OS1, OS2, OS4, OS5, PS2, PS3, PS4, PS5 was used for adipogenic, osteogenic and chondrogenic differentiation tests. ^4^ Material from OS1-OS5 and PS1-PS5 was used for flow cytometry and quantitative PCR analysis.

**Supplementary Table S4**

| **Categories** | **Diseases or Functions Annotation** | **p-Value^a^** | **Predicted Activation State** | **Activation z-score** | **Number of involved Molecules** |
| --- | --- | --- | --- | --- | --- |
| Cellular Movement | Cell movement | 1,76E-15 | Decreased | -2,610 | 79 |
| Cellular Movement | Migration of cells | 8,28E-16 | Decreased | -2,531 | 74 |
| Cellular Assembly and Organization, Cellular Function and Maintenance | Organization of cytoskeleton | 1,13E-04 | Decreased | -2,283 | 33 |
| Cellular Assembly and Organization, Cellular Function and Maintenance | Organization of cytoplasm | 5,41E-04 | Decreased | -2,283 | 35 |
| Cellular Movement | Invasion of cells | 1,81E-15 | Decreased | -2,227 | 53 |

**Supplementary Table S4:** Top functions and diseases predicted to be altered in ASCs after FBS deprivation compared to ASCs cultured with standard condition media with FBS. Z-score is applied in IPA to reduce the chance that random data will produce significant prediction. It identifies functions with the strongest prediction for increase (positive z-score) or decrease (negative z-score). Values of z- score ≤−2 or ≥ 2 are considered significant.
^a^ Fisher’s exact test was implemented to measure the likelihood that the association between the differentially expressed genes and a given pathway is not due to random chance; the pathways with p-value>10E-05 should be interpreted with caution.

**Supplementary Table S6**

| Gene | Forward primer^1^ | Reverse primer^2^ | Product | Length |
| --- | --- | --- | --- | --- |
| *ITGAM* | CCCATTACTACGAGCAGACCC | CCCCTGGGCTCCTACAGTCAG | NM001145808.1  NM000632.3 | 385 bp  382 bp |
| *ITGA6* | GTGGCTATTCTCGCTGGGAT | TGTAAGTCAGCCACGCCAAA | NM001079818.2  NM000210.3 | 492 bp  622 bp |
| *PODXL* | CCTCCACAGCCACAGCTAAA | CCTGTGAGGTTCAGGACGAG | NM001018111.2  NM005397.3 | 682 bp  586 b |

**Supplementary Table S6:** Primers used for *ITGAM, ITGA6* and *PODXL* isoform analysis with amplification products and their length. Forward primer **-** sequence of forward primer 5'-3'; Reverse primer – reverse primer sequence 5'-3', ^1^ M13 forward sequencing adapter 5’-GTAAAACGACGGCCAGT-3’ was attached to primer sequence, ^2^ M13 reverse sequencing adapter 5’-CAGGAAACAGCTATGAC-3’ was attached to primer sequence.

**SUPPLEMENTARY FIGURES**

**Supplementary Figure S1**

**
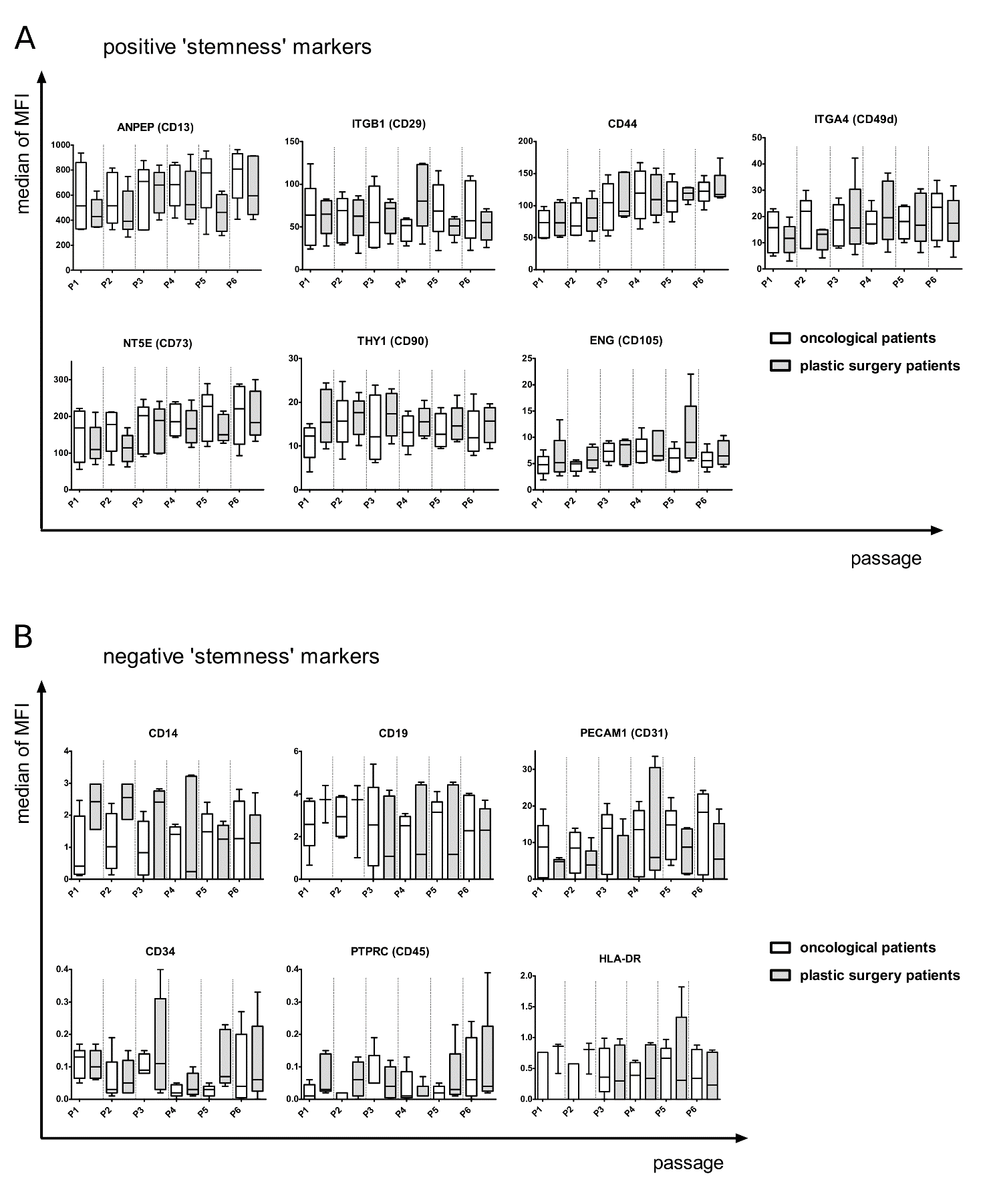
**

**
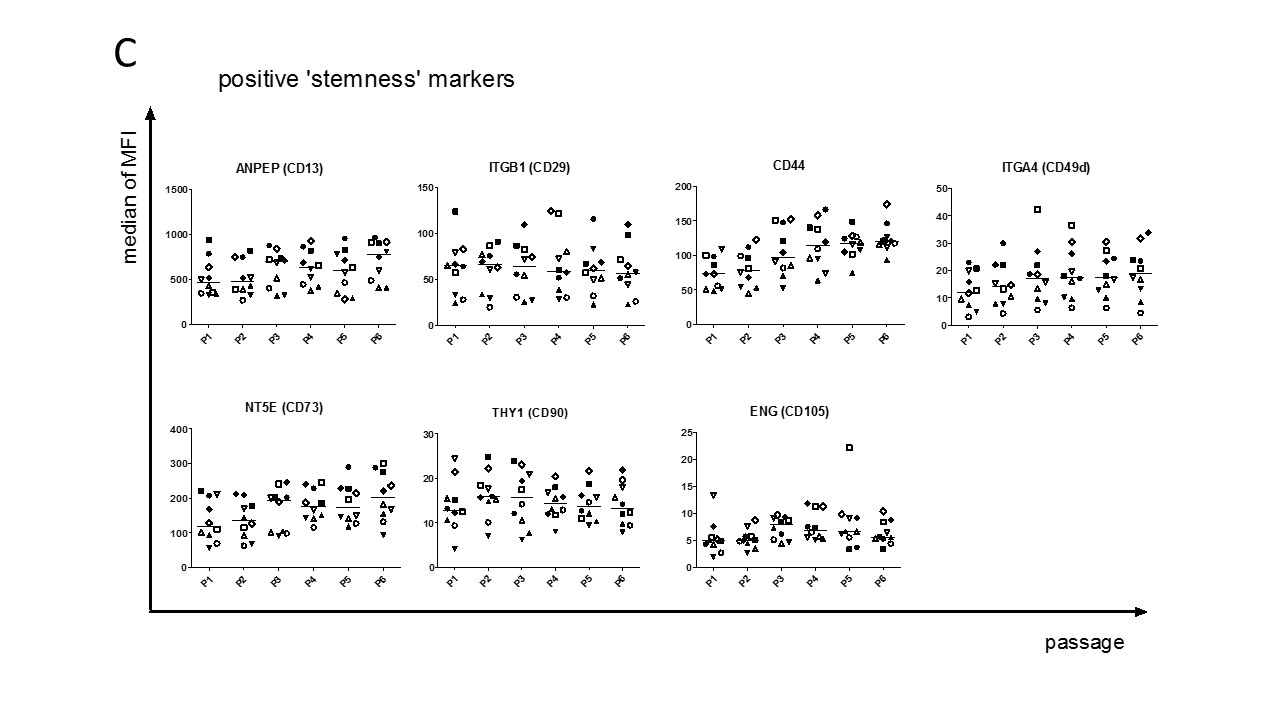
**

**
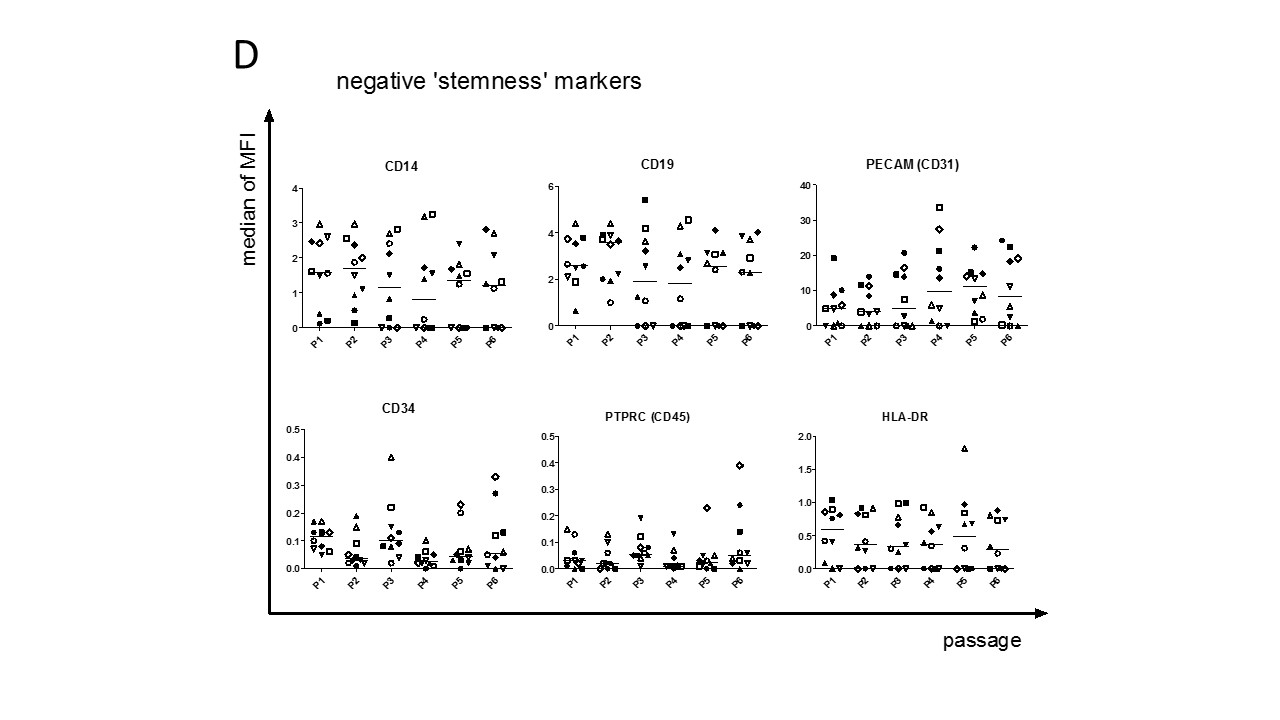
**

**Supplementary Figure S1**: The flow cytometry analysis of the differences between analyzed groups – oncological patients vs. plastic surgery patients according to the presence of positive (A) and negative (B) ASCs markers. The X axis represents the passage number (from P1 to P6), whereas Y axis shows the median of MFI (mean fluorescence intensity). Comparison of the phenotypic parameters between two groups (oncological and plastic surgery patients) were performed with the Mann-Whitney U test. No statistically significant differences were observed between these two groups of patients (the difference was considered significant when p-value was ≤ 0.01). Center lines denote the median, box limits indicate the 25^th^ and 75^th^ percentiles; whiskers represent the maximum and minimum of the acquired values.
The phenotypic analysis of cultured ASCs. Flow cytometry assessment of both positive (C) ASCs markers: ANPEP (CD13), ITGB1 (CD29), CD44, ITGA4 (CD49d), NT5E (CD73), THY1 (CD90) and ENG (CD105); and negative (D) surface markers: CD14, CD19, PECAM (CD31), CD34, PTRC (CD45), HLA-DR. The X axis represents the passage number (from P1 to P6), whereas the Y axis displays the mean fluorescence intensity (MFI) of cells expressing particular surface marker. Center lines denote the median of MFI (dots represent data from each patient).

**Supplementary Figure S2**

**
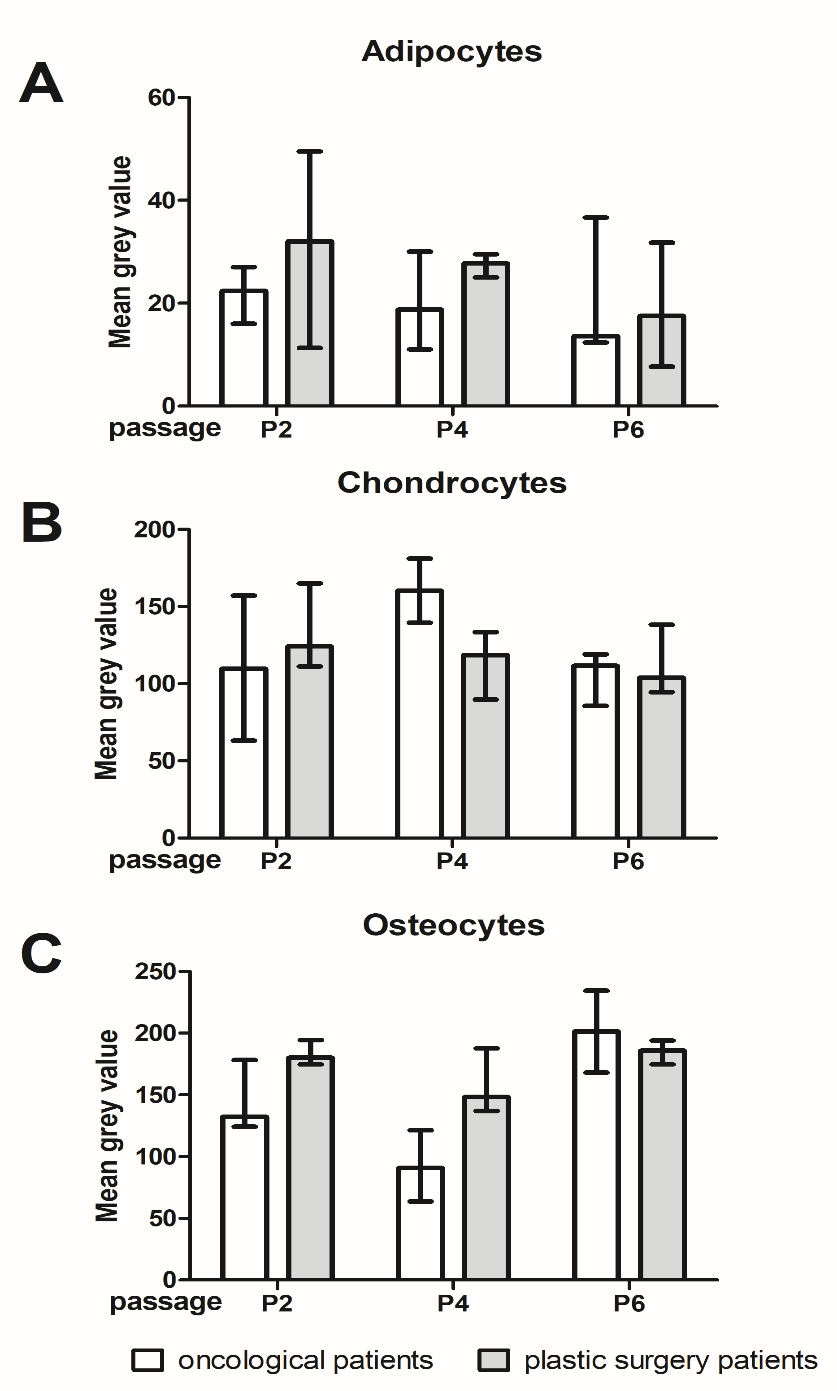
**

**Supplementary Figure S2:** Quantitative analysis of the multilineage differentiating potential of oncological and plastic surgery patients ASCs. Oil-red O (A), Alcian blue (B), Alizarin red (C) positive staining areas collected as mean grey values of photos analyzed by ImageJ v. 1.52 (U.S. National Institutes of Health; <http://rsb.info.nih.gov/ij/>). The threshold was established based on the control images (mean grey value from control images – undifferentiated cells). Final values are presented as medians with range from 4 oncological and 4 plastic surgery patients. Comparison between two groups (oncological and plastic surgery patients) was performed with the Mann-Whitney U test. No statistically significant differences were observed between these two groups of patients (p-value ≤ 0.01).

**Supplementary Figure S3**

**Supplementary Figure S3:** Cumulative population doublings of ASCs from oncological (white) and plastic surgery patients (grey) cultivated in standard conditions up to the 6^th^ passage (median values with ranges representing variation between patients). Population doubling was determined for each passage and added to the population doublings of the previous passages. Comparison between two groups (oncological vs plastic surgery patients) was performed with the Mann-Whitney U test. No statistically significant differences were observed between these two groups of patients (p-value ≤ 0.01).

**Supplementary Figure S4**

**Supplementary Figure S4**: RNA-seq biological pentaplicate results comparison of ASCs markers expression with FBS-deprived medium (blue bars) and with FBS supplemented condition (orange bars). Gene expression is presented in FPKM- Fragments Per Kilobase of transcript per Million mapped reads values. Data features two panels for positive (A) and negative (B) ASCs markers. Statistically significant change is indicated with star sign (*).

**Supplementary Figure S5**

**
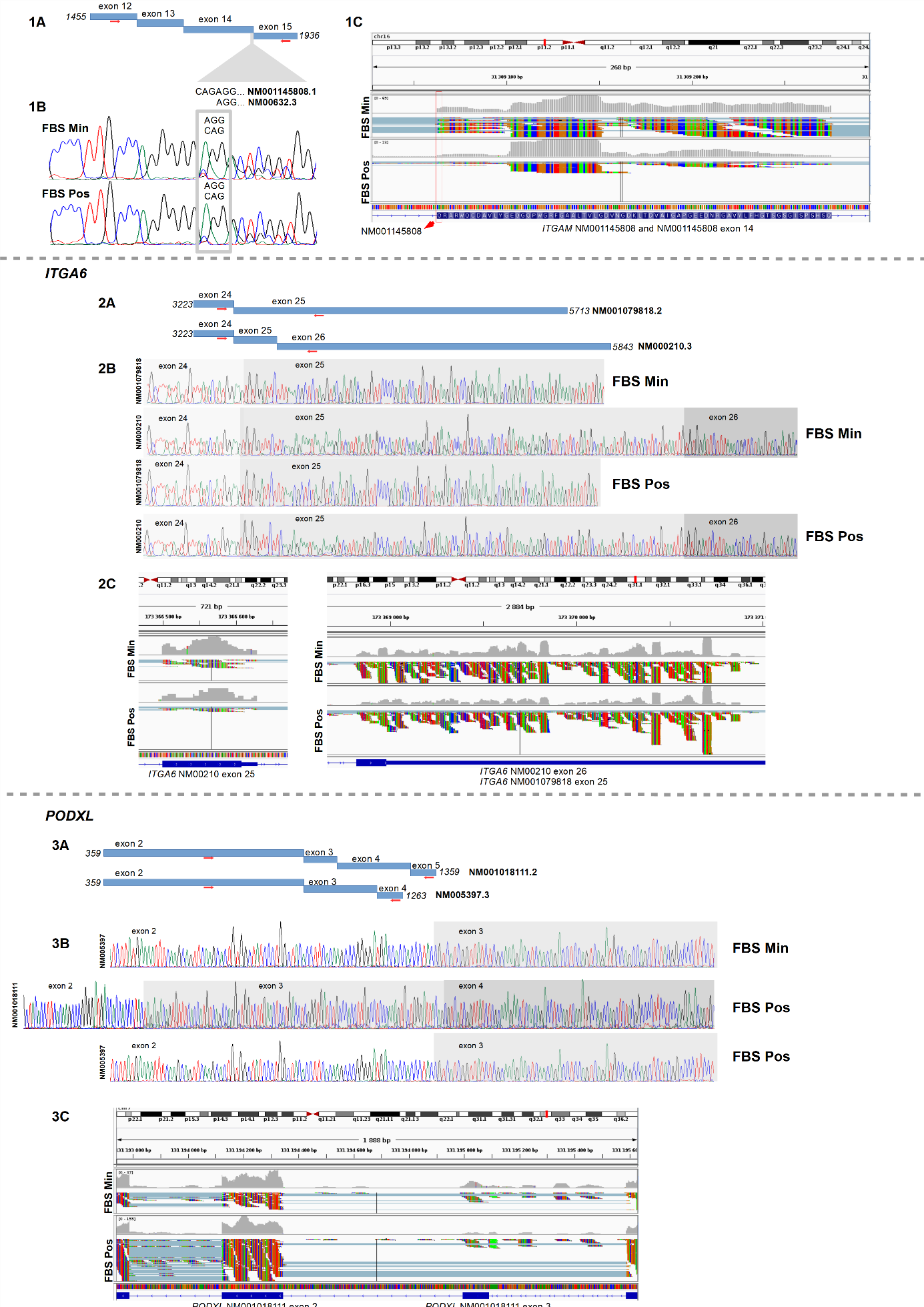
**

**Supplementary Figure S5**: Sanger sequencing confirmation of *ITGAM (CD11b)*, *ITGA6* and *PODXL* isoforms for ASCs cultured in absence and presence of FBS (labelled as FBS Min and FBS Pos)*.* Schematic diagrams (blue, marked as 1A, 2A and 3A) show the regions that distinguish isoforms as detected in RNA-seq experiments. Location of forward and reverse primers used for sequencing areas indicated with red arrows. (**1A**) Two existing isoforms of *ITGAM (CD11b)* (var. 1 NM001145808.1 and var. 2 NM00632.3) differ only by one codon in exon 14, **(1B)** Sanger sequencing confirmation of the presence of both isoforms in sample PS9 in ASCs cultured without and with FBS, **(1C)** IGV visualization of exon 14 (merged BAM files for 5 replicates) show the coverage for both existing isoforms, albeit with sparse NGS reads for isoform NM001145808. **(2**A) Two out of three known *ITGA6* isoforms NM001079818.2 (var. 1) and NM000210.3 (var. 2), as detected by RNA-seq, of which var. 2 has one extra 129 bp exon from position 3349 to 3478, **(2B)** Sanger sequencing confirmation of the presence of both isoforms in sample PS8 in FBS Min and FBS Pos conditions, **(2C)** IGV visualization of merged BAM files for 5 replicates show lower coverage for NM000210 (unique exon 25) cultured without and with FBS, and higher coverage for exons 25/26 that are shared by both *ITGA6* isoforms. **(3A)** Isoform NM001018111.2 (var. 1) of *PODXL*  differs from NM005397.3 (var. 2) with an extra exon (96 bp) at position 965, **(3B)** Sanger sequencing of this region showed the presence of only NM005397.3 variant in sample PS9 cultured in absence of FBS and presence of both isoforms while grown with FBS, **(3C)** IGV visualization of merged BAM files for 5 replicates show higher coverage for exon 2 shared by NM005397 and NM001018111 for ASC without and with FBS, and very low coverage for unique exon 3 of NM001018111.
